# Comparison of Parameters for Assessment of Carotid Stiffness and Their Association with Carotid Atherosclerosis in Rural Australian Adults: A Pilot Study

**DOI:** 10.3390/jcm12082935

**Published:** 2023-04-18

**Authors:** Marjana Petrova, Alex Gavino, Yujie Li, Craig S. McLachlan

**Affiliations:** Centre for Healthy Futures, Torrens University Australia, Sydney, NSW 2010, Australia

**Keywords:** carotid stiffness, carotid plaques, carotid ultrasound, echo-tracking, rural population

## Abstract

Carotid stiffness has been associated with the development and progression of carotid artery disease and is an independent factor for stroke and dementia. There has also been a lack of comparison of different ultrasound-derived carotid stiffness parameters and their association with carotid atherosclerosis. This pilot study aimed to investigate the associations between carotid stiffness parameters (derived via ultrasound echo tracking) and the presence of carotid plaques in Australian rural adults. In cross-sectional analyses, we assessed forty-six subjects (68 ± 9 years; mean ± SD) who underwent carotid ultrasound examinations. Carotid stiffness was assessed by a noninvasive echo-tracking method, measuring and comparing multiple carotid stiffness parameters, including stroke change in diameter (ΔD), stroke change in lumen area (ΔA), β- stiffness index, pulse wave velocity beta (PWV-β), compliance coefficient (CC), distensibility coefficient (DC), Young’s elastic modulus (YEM), Peterson elastic modulus (Ep), and strain. Carotid atherosclerosis was assessed bilaterally by the presence of plaques in the common and internal carotid arteries, while carotid stiffness was assessed at the right common carotid artery. β-stiffness index, PWV-β, and Ep were significantly higher (*p* = 0.006, *p* = 0.004, *p* = 0.02, respectively), whilst ΔD, CC, DC, and strain were lower among subjects with carotid plaques (*p* = 0.036, *p* = 0.032, *p* = 0.01, *p* = 0.02, respectively) comparing to subjects without carotid plaques. YEM and ΔA did not significantly differ among the groups. Carotid plaques were associated with age, history of stroke, coronary artery disease, and previous coronary interventions. These results suggest that unilateral carotid stiffness is associated with the presence of carotid plaques.

## 1. Introduction

The aging process is associated with an increased risk for vascular stiffness [1,2]. Precise noninvasive methods to measure stiffness in the carotid artery have emerged and can be conducted in clinical settings [3,4]. Several smaller and larger scale studies suggest that individuals with pre-existing cardiovascular diseases (sub-clinical or overt) have increased indices of carotid stiffness compared to individuals without established cardiovascular disease [5,6,7]. Carotid stiffness has also been suggested to predict cardiovascular events and all-cause mortality [8]. Greater carotid stiffness measured by beta (β) stiffness, Young’s elastic modulus, distensibility coefficient, and compliance coefficient has also been associated with the incidence of stroke, independently from other covariates such as age, sex, and recognized common population cardiovascular risk factors [9].

The association between arterial stiffness and localized atherosclerosis may be explained by the natural progression of atherosclerosis or an increase in pulse pressure. In the early stages of atherosclerosis, arterial elasticity and compliance decrease due to the degradation of elastic fibers and increased collagen deposition in the arterial wall. As a result, arterial stiffness increases before any visible changes appear in the vascular wall structure [10]. Moreover, stiffening in the carotid arteries may lead to increased pulse pressure and flow pulsatility, which can induce endothelial dysfunction, a precursor to the development of atherosclerosis [11]. Hence, the noninvasive determination of arterial stiffness may be useful in the evaluation of early-stage carotid disease.

Carotid ultrasound and automated software for wall tracking [3,4] are some of the several imaging modalities that allow accurate measurement of local carotid stiffness, even though the methods and reference values of carotid stiffness parameters have not been standardized to use in routine clinical practice.

While associations between arterial stiffness and atherosclerotic disease have been explored, most studies have examined carotid stiffness on underlying atherosclerotic pathology in the same corresponding carotid site of stiffness. Hence, it remains unknown whether stiffness in one segment of the carotid can be associated with remote carotid plaque burden. Additionally, while rural populations have an increased risk for stroke, the role of arterial stiffness and risk for carotid plaques in those cohorts have been rarely reported [12]. Our study aims to evaluate the association between various carotid stiffness parameters from a wall-tracking software platform and the presence of carotid plaque in both carotid arteries among a community sample from the Ararat rural population.

## 2. Materials and Methods

### 2.1. Study Settings

The present sub-study was a cross-sectional analysis of a prospective community study conducted among rural residents of Ararat in Victoria, Australia, aged 35 and older. The participant recruitment and study design details have been described previously [13]. Between March 2022 and July 2022, forty-five subjects completed follow-up health assessments and underwent carotid ultrasound examinations, and one new participant was enrolled in the study, All study subjects included in these analyses were Caucasian and aged between 48 to 81 years.

### 2.2. Ethical Consideration

All subjects provided written informed consent before completing the health survey and the health assessments. The Ararat Health Study was approved by the Human Research Ethics Committee of Ballarat Health Service and St. John of God Hospital.

### 2.3. Health Assessments

Health assessments included anthropometric measurements, blood pressure (BP), and 12-lead electrocardiogram (ECG). Mean arterial pressure (MAP) was calculated as 2/3 diastolic blood pressure (DBP) + 1/3 systolic blood pressure (SBP).

### 2.4. Carotid Artery Ultrasound

In addition to the abovementioned follow-up health assessments, a carotid ultrasound examination was performed for this study. All imaging was performed using the Phillips affinity 70 ultrasound equipment and an eL18-4 ultra-broadband linear transducer with PureWave crystal technology.

Carotid artery images and cine loops were acquired using standardized protocols in accordance with the recommendations of the Manheim statements and recommendations [14].

Carotid ultrasound examinations were performed in a comfortable room with a temperature of 21–22 °C. All subjects were placed in a relaxed supine position with the neck tilted 45° opposite to the scanned side. Bilateral carotid arteries (including the common carotid, carotid bifurcation, and internal carotid arteries) were scanned in transverse and longitudinal sections.

### 2.5. Intima-Media Thickness and Carotid Stiffness Parameters

First, a clear longitudinal image in B-mode was obtained from the right common carotid artery. Then, 5–10 s (150–301 frames) cine loops were recorded and transferred to an ultrasound database for further offline analyses. Finally, all cine loops were reviewed to select loops that met the critical optimization criteria: precise near and far wall intima-media, clear lumen, a minimum of 3 cardiac cycles, and straight vessel.

Carotid intima-media thickness (IMT), maximum lumen diameter (Ds), and stroke change in diameter (ΔD) were measured at least 5–10 mm below the carotid bifurcation in a region free of carotid plaque using an automatic software platform for ultrasound imaging analyses (CAROLAB v5.0) [15]. Carotid IMT analyzed by CAROLAB was expressed as the mean of the maximum IMT measured in each carotid segment (in 3–6 cardiac cycles).

The following carotid stiffness parameters for subsequently calculated:

Stroke change in the lumen area (ΔA)

ΔA = π (Ds^2^ − Dd^2^)/4 (mm^2^)

β stiffness parameter

β = ln (SBP/DBP)/[ΔD/Dd] (unitless)

Cross-sectional distensibility coefficient (DC)—relative change in lumen area during systole for a given pressure change

DC= 2ΔD/ΔPDs (Kpa 10^−3^)

Cross-sectional compliance coefficient (CC)—absolute change in lumen area during systole for a given pressure change

CC = (π × Ds × ΔD)/(2 × ΔP) (10^−7^ m^2^ Kpa^−1^)

Peterson’s elastic modulus (Ep)—the inverse of distensibility coefficient: the pressure change directing an increase in relative lumen area

Ep = (SBP − DBP)/[(Ds−Dd)/Dd] (Kpa)

Young’s elastic modulus (YEM)—elasticity of the arterial wall material, considering the thickness of the arterial wall

YEM = (ΔP ×Dd)/(ΔD × IMT) (mmHg/mm)

Strain

Strain = ΔD/Dd × 100 (%)

One-point pulse wave velocity β (PWV-β)—calculated from the time delay between two adjacent distensions waveforms from the water hammer equation with the usage of β-stiffness parameter

PWV-β = √ (β × Pd/2 × ρ) (m s^−1^),

where Ds = systolic diameter; Dd = diastolic diameter; IMT = intima-media thickness; SBP = systolic blood pressure, DBP = diastolic blood pressure; ΔP = pulse pressure; ρ- blood density = 1050 kg/m^3^

### 2.6. Carotid Plaque Measurement

The presence of atherosclerotic plaques in the carotid arteries was determined by evaluation of the ultrasound images of the common, internal, and bifurcation sites of the left and right carotid arteries. According to the Manheim consensus [14], plaques were defined as structures encroaching into the arterial lumen of at least 0.5 mm or 50% of the surrounding IMT value or demonstrated a thickness ≥ 1.5 mm as measured from the intima-lumen interface to the media-adventitia interface.

### 2.7. Statistical Analyses

Continuous data were expressed as means and standard deviations or as medians and interquartile ranges. Categorical data were expressed as total counts and percentages. The normality of data was assessed using the Shapiro—Wilk test. We used an independent *t*-test and Mann—Whitney U test to investigate differences among carotid stiffness parameters and risk factors in the groups with and without carotid plaques. Categorical variables were compared by Chi-square test (χ^2^ test). The results were considered significant at *p* < 0.05. All data were analyzed using IBM SPSS Statistics version 28.01.

### 2.8. Reproducibility

Intra-observer reproducibility was assessed by two consecutive offline measurements (at different time points) of IMT, Ds, and ΔD in 15 randomly selected subjects. Intra-observer reproducibility was expressed by calculating the intraclass correlation coefficient (ICC).

## 3. Results

### 3.1. Subject Characteristics

Table 1 shows the demographic characteristics of the study subjects. The mean age was 67.91 ± 8.72 years, with 30 (65.2%) subjects falling in the 55–74 years age range. Among the 46 subjects, there were 27 females (58.7%) and 19 males (41.3%).

### 3.2. Carotid Stiffness Parameters in Subjects with and without Plaque

Patients were divided into two groups depending on the presence of plaque. Plaques were present on either left or right common and internal carotid arteries. Fifteen subjects had a carotid plaque in at least one carotid bed—eight had bilateral carotid plaques, four had plaques on the left, and three had plaques on the right (Table 1). One subject was excluded from the comparative analyses, as we were unable to perform an estimation of the stiffness parameters due to the low quality of the ultrasound cine loops. We evaluated the difference in mean or median (sd or IQR) in the following markers of carotid stiffness—ΔD (mm), ΔA (mm^2^), β, PWV-β (m/s^−1^), CC (10^−7^ m^2^ Kpa^−1^), DC (10^−3^ Kpa), YEM (mmHg/mm), Ep (Kpa), Strain (%)—as well as the systolic and diastolic carotid diameter and the IMT. Results are presented in Table 2.

In subjects with no carotid plaques, the stroke change in diameter (ΔD), distensibility coefficient (DC), and strain (%) were significantly higher compared to the group with atherosclerotic plaques, i.e., ΔD (*p* < 0.036); DC (*p* = 0.01); Strain (*p* = 0.02). The following stiffness parameters—β-index, PWV-β, and Peterson Elastic Modulus (Ep)—were significantly lower in subjects without plaque compared to subjects with atherosclerotic plaques, as follows: β (*p* = 0.006); PWV-β (*p* = 0.04); Ep (*p* = 0.02). We also found that IMT was significantly higher among subjects with carotid plaques (*p* = 0.01). In contrast, YEM did not significantly differ among the groups. Mean systolic (Ds) and diastolic (Dd) carotid diameters were higher in the plaque group; however, these differences were not statistically significant.

### 3.3. Carotid Plaque and Traditional Risk Factors

Subjects in the carotid plaque group were significantly older compared to the group without carotid plaque (*p* < 0.01). Both groups were comparable in gender, SBP, DBP, PP, BMI, waist circumference, hypertension, and lipid status. In subjects with carotid plaque, the percentage of stroke, myocardial infarction (MI), and coronary intervention were higher compared to the group without plaque (*p* = 0.01, *p* = 0.03, and *p* = 0.003, respectively). Detailed characteristics of cardiovascular risk factors are presented in Table 3.

### 3.4. Intra-Observer Reproducibility

Intra-observer reliability was excellent for IMT (Reading 1 (0.82 ± 0.19, mean ± SD); Reading 2 (0.88 ± 1.19, mean ± SD); ICC = 0.94); good for Ds (Reading 1 (6.25 ± 0.58, mean ± SD); Reading 2 (6.68 ± 0.82), ICC = 0.78); and good for ΔD (Reading 1 (0.52 ± 0.11, mean ± SD); Reading 2 (0.55 ± 0.14., mean ± SD), ICC = 0.76).

## 4. Discussion

Our study demonstrates, for the first time, an ability to comprehensively compare carotid ultrasound parameters using various validated formulae of stiffness and their association with the presence of carotid plaques. Stiffness parameters that we included were absolute distensibility (ΔD), stroke change in absolute diameter (ΔA), cross-sectional compliance coefficient (CC), distensibility coefficient (DC), β stiffness index, βPWV, Peterson elastic modulus (Ep), and strain. Of these stiffness parameters, we found β, PWV-β, and Ep were significantly higher, whilst absolute distensibility, distensibility coefficient, and strain were lower among subjects with carotid plaques. These results are consistent with individual studies examining carotid distensibility, compliance, and stiffness indices in atherosclerotic patients [16,17,18]. Studies show that carotid distensibility is significantly associated with the presence and severity of atherosclerosis [19,20]. The carotid distensibility coefficient and Young’s elastic modulus were related to symptomatic carotid disease [21,22], and these correlate with the degree of atherosclerosis [23]. Giannattasio et al. [16] found that arterial distensibility was markedly lower in the stenotic internal carotid artery compared to the plaque-free contralateral internal carotid artery. However, it was also found to be lower in the ipsilateral common carotid artery, indicating the effect of stiffness beyond the actual plaque site. It is essential to highlight that in our study, we measured the stiffness in the right common carotid artery; however, the presence of plaque was determined across both the left and right common and internal carotid arteries.

The presence of atherosclerotic plaques has been associated with arterial stiffness in studies investigating different vascular beds. For example, atherosclerotic plaques in the aorta were associated with decreased aortic distensibility [24]. The augmentation index of the aorta was also found to be significantly associated with peripheral artery disease [25].

A few possibilities for the determined association between carotid stiffness and carotid atherosclerosis can be hypothesized. One possibility is that the atherosclerotic process leads to an increase in arterial stiffness by triggering hemodynamic changes and vascular cell dysfunction, promoting atherosclerosis [26]. An alternative possibility is that the increased arterial stiffness precedes the damage of the vascular wall and then causes atherosclerosis. Without the buffering capacity, stiff arterial walls may be exposed to increased intraluminal stress related to increased pulse pressure [27]. A third possibility is that both mechanisms are valid. Atherosclerosis may be a result of increased stiffness, but then it may further increase arterial stiffness in advanced stages. Finally, another possibility is that these processes may be independently processed but may happen simultaneously in the arterial wall without a specific temporal or causational relationship.

Despite the availability of accurate imaging modalities for evaluating carotid stiffness, there are no current standardized methods and parameters for evaluating carotid stiffness, and their application in clinical practice is limited [28].

In our study, the presence of carotid plaques was associated with older age and a history of previous stroke, myocardial infarction, and previous coronary interventions. Age is a common risk factor for atherosclerotic disease, and a close relationship between coronary and carotid atherosclerosis has been examined in symptomatic and asymptomatic patients [29,30]. Johnses et al. [31] reported that carotid plaque is a strong risk factor and predictor of myocardial infarction, while Novo et al. [32] reported a high presence of asymptomatic plaques in patients with three-vessel disease. Such evidence, along with our study findings, aligns with the hypothesis that atherosclerosis is a systemic disease and is often not limited to one single vascular bed.

To the best of our knowledge, our study is the first to simultaneously investigate multiple carotid stiffness ultrasound parameters published in the literature and their associations with carotid atherosclerosis.

Populations living in rural areas have an increased burden of carotid plaque coronary artery disease and cardiovascular risk factors compared to urban populations [33,34]. However, there is a paucity of data to estimate the global burden of carotid atherosclerosis among rural populations [21]. Increased intima-media thickness and carotid plaques are well-established parameters for diagnosing subclinical and clinical carotid artery disease; however, screening in the general population is still under debate [35,36]. Other ultrasound imaging modalities, such as the assessment of carotid plaque burden using 3D ultrasound imaging, have been useful for the assessment of carotid plaque burden, progression, risk stratification, and evaluation of new risk factors [37]. However, despite the significant prognostic implications and high accuracy, 3D modality remains a niche area, mainly due to the high cost of the probes and the need for specialized vascular laboratories.

While we can hypothesize that carotid stiffness may be associated with plaque risk in the other vascular territory, such as the right common carotid, we, however, could not measure stiffness in the contralateral or other vascular beds. Hence, we can say that when we find stiffness in one carotid artery with non-plaque deposits, it is possible to observe plaques in the opposite carotid artery. This may be explained by remote signaling, or in fact, the other bed may also be stiff; if the latter is the case, we currently do not have a plausible explanation. Finally, as our study was cross-sectional by design, causality cannot be inferred.

Our study has a few perceived limitations that warrant discussion. Firstly, our study has a moderate number of participants. On the other hand, this study was pre-planned as a pilot study to assess methodological assessment on the basis of which stiffness parameter is best associated with carotid plaque burden. Thus, the study had a sufficient sample size to explore these associations. The second perceived limitation is that this study focused on plaque burden and not stenosis. The study was not pre-designed to consider variances in carotid artery stenosis, apart from high-grade stenosis. Indeed, increased aortic stiffness has been associated with the presence of higher-grade carotid stenosis > 50% [38]. It should be noted that none of our participants had high-grade carotid stenosis.

Our study shows that carotid distensibility and stiffness indices are associated with the presence of carotid atherosclerosis. Future studies with more standardized methods of measurement and validation of carotid stiffness may contribute to greater clinical implementation of ultrasound imaging parameters into clinical practice and assessment of early carotid atherosclerosis. The assessment of carotid stiffness was dependent on the type of ultrasound parameter used to reliably reflect sub-clinical carotid atherosclerosis.

## Figures and Tables

**Table 1:** Demographic characteristics of the study subjects.

Demographic Characteristics (n = 46)	Mean ± SD or n (%)
Age (years)	67.91 ± 8.72
35–54	4 (8.7)
55–74	30 (65.2)
>75	12 (26.1)
Gender	
Male	19 (41.3)
Female	27 (58.7)
Smoking	
Current	2 (4.3)
Former	17 (37)
Never smoked	27 (58.7)
Living in Ararat (years)	31.18 ± 17.88
Anthropometric characteristics	
BMI (kg/m^2^)	27.4 ± 4.74
Normal	17 (37)
Overweight	20 (43.5)
Obese	9 (19.6)
Height (cm)	167.50 ± 8.58
Weight (kg)	77.08 ± 15.50
Waist circumference (cm)	
Male	96.84 ± 11.43
Females	89.56 ± 12.4
Self-reported medical conditions	
Hypertension	19 (41.3)
Antihypertensive medications	15 (32.6)
Dyslipidemia	24 (52.2)
Antilipid medications (statins)	9 (19.6)
Diabetes type 2	4 (8.7)
Cardiovascular disease	7 (15.2)
Hemodynamic parameters	
SBP (mmHg)	130.13 ± 13.23
DBP (mmHg)	75.91 ± 9.21
MAP (mmHg)	93.99 ± 9.36
PP (mmHg)	54.21 ± 11.32
Resting HR (beats per min, bpm)	64.48 ± 10.60
QRS duration (ms)	102.02 ± 22.59
Carotid ultrasound parameters	
IMT (mm)	0.75 ± 0.16
Carotid plaques	15 (32.6)
Carotid plaques, unilateral (Left CCA and ICA)	4 (26.6)
Carotid plaques, unilateral (Right CCA and ICA)	3 (20)
Carotid plaques (bilateral)	8 (53.3)

CCA—common carotid artery; ICA—internal carotid artery; SBP = systolic blood pressure; DBP = diastolic blood pressure; MAP = mean arterial pressure; PP = pulse pressure

**Table 2:** Carotid stiffness parameters in subjects with or without carotid plaque.

	Plaques, No (n = 31)	Plaques, Yes (n = 14)	
	Mean ± SD	Mean ± SD	*p*-Value
Ds (mm)	5.93 ± 0.54	6.01 ± 0.57	0.63
Dd (mm)	5.44 ± 0.52	5.59 ± 0.53	0.41
ΔD (mm)	0.48 ± 0.91	0.41 ± 0.08	0.036 *
ΔA (mm^2^)	4.33 ± 1.00	3.86 ± 1.47	0.16
β stiffness	6.09 ± 1.53	7.73 ± 2.15	0.006 *
PWV β (m/s^−1^)	5.36 ± 0.59	5.9 9± 0.71	0.004 *
CC (mm^2^ Kpa^−1^)	6.59 ± 1.58	5.76 ± 7.97	0.032 *
DC (10^−3^ Kpa)	23.80 ± 7.91	19.94 ± 0.94	0.01 *
YEM (mmHg/mm)	883.97 ± 284.51	963.49 ± 333.31	0.53
Ep (Kpa)	(613.97, 155.24) **	(749.01, 239.39) **	0.02 *
Strain, %	8.82 ± 1.84	7.56 ± 1.49	0.02 *
IMT (mm)	0.71 ± 0.13	0.84 ± 0.19	0.01 *

Legend: Ds = systolic diameter; Dd = diastolic diameter; ΔD = stroke change in diameter; ΔA = stroke change in lumen area; CC = compliance coefficient; DC = distensibility coefficient; YEM = Young’s elastic modulus; Ep = Peterson’s Elastic modulus, * *p* < 0.05, ** median (IQR).

**Table 3:** Cardiovascular risk factors among subjects with and without carotid plaque.

	Plaques, No (n = 31)	Plaques, Yes (n = 15)	
	Mean ± SD or n (%)	Mean ± SD or n (%)	*p*-Value
Age	65.71± 8.69	72.47± 7.029	0.01 *
Sex, males	9 (32.3)	10 (60.0)	0.73
SBP	130.84 ± 13.16	133.93 ± 15.87	0.5
DBP	80.23 ± 8.40	77.27 ± 7.06	0.12
PP	50.7 1± 11.93	56.67 ± 12.34	0.12
MAP	96.16 ± 8.50	96.20 ± 8.98	0.9
BMI	27.55 ± 5.23	27.08 ± 3.66	0.75
Waist circumference	91.90 ± 13.48	93.93 ± 10.13	0.61
Self-reported conditions			
Hypertension	9 (29.0)	6 (40.0)	0.45
Dyslipidemia	15 (62.5)	9 (37.5)	0.46
Diabetes	2 (6.5)	2 (13.3)	0.43
Stroke	0 (0)	3 (27.9)	0.01 *
Myocardial infarction	0 (0)	2 (13.3)	0.03 *
Coronary intervention	0 (0)	4 (26.7)	0.003 *

* p < 0.05; SBP = systolic blood pressure; DBP = diastolic blood pressure; MAP = mean arterial pressure; PP = pulse pressure

## Data Availability

Sharing of data is unavailable due to privacy or ethical restrictions.
